# Gestaltungs- und Evaluationsmöglichkeiten von Multimediaanwendungen zur Unterstützung der Betreuung von Menschen mit leichter Demenz

**DOI:** 10.1007/s00391-023-02280-2

**Published:** 2024-01-24

**Authors:** Alexa von Bosse, Alexander Bejan, Max Wessel, Christophe Kunze

**Affiliations:** Institut Mensch Technik und Teilhabe, Fakultät Gesundheit, Sicherheit, Gesellschaft, Hochschule Furtwangen, Robert-Gerwig-Platz 1, 78120 Furtwangen im Schwarzwald, Deutschland

**Keywords:** Touchbasierte digitale Technologien, Digitale Pflegeanwendung, Demenzielle Symptome, Häusliche Versorgung, Pflegende Angehörige, Touch-based digital technologies, Digital care application, Demential symptoms, Home care setting, Caregiving relatives

## Abstract

**Hintergrund:**

Zur kognitiv-körperlichen Aktivierung von Menschen mit Demenz (MMD) im stationären Setting stehen vielfältige, zielgruppenspezifisch konzipierte touchbasierte Multimedia-Anwendungen zur Verfügung, die auch tatsächlich in der Praxis genutzt werden. Im Vergleich dazu ist der Einsatz vergleichbarer Anwendungen in der Häuslichkeit bislang gering ausgeprägt.

**Ziel und Methodik:**

Die vorliegende Studie untersucht anhand von 11 leitfadengestützten Expert*inneninterviews sowohl Gestaltungsoptionen und Anforderungen an Anwendungsinhalte als auch Anforderungen an Studiendesigns zu Wirksamkeitsnachweisen touchbasierter Multimedia-Anwendungen für MMD und ihre pflegenden Angehörigen (PA).

**Ergebnisse:**

Es zeigt sich eine große Übereinstimmung bezüglich allgemeiner Anforderungskriterien an touchbasierte Multimedia-Anwendungen – die Akzeptanz ist in hohem Maße von Nutzungskompetenzen, Nutzungspraktiken, Erfahrungen sowie Anreizstrukturen abhängig. Auf der anderen Seite liegen heterogene Meinungen zur inhaltlich-strukturellen Ausgestaltung touchbasierter Multimedia-Anwendungen für MMD vor. Lebensqualität wird als elementarer Evaluationsparameter von Multimedia-Anwendungen genannt.

**Diskussion:**

Individuell stark variierende Lebensumstände von MMD und ihren PA sowie mangelnde Zugangsmöglichkeiten und fehlende Passgenauigkeit der Multimedia-Anwendungen können ursächlich für ihren bislang geringen Einsatz insbesondere in der Häuslichkeit sein. Tagesabhängige Leistungsfähigkeiten und individuelle Krankheitsverläufe stellen besondere Anforderungen an die wissenschaftliche Evaluation und an Wirksamkeitsnachweise touchbasierter Multimedia-Anwendungen dar.

**Zusatzmaterial online:**

Zusätzliche Informationen sind in der Online-Version dieses Artikels (10.1007/s00391-023-02280-2) enthalten.

## Abstract (en)

**Background:**

In order to cognitively and physically activate people with dementia (MMD) in institutional settings, there are numerous touch-based multimedia applications specifically designed for this target group that are actually being used in practice. In contrast, the use of similar applications in domestic settings has been relatively limited.

**Aim and method:**

This study is based on 11 expert interviews guided by questions and examines design options and requirements for application content as well as requirements for study designs to provide evidence of the effectiveness of touch-based multimedia applications for MMD and their caregiving relatives (PA).

**Results:**

There is a high degree of consensus regarding the criteria for requirements for touch-based multimedia applications. Acceptance is highly dependent on user competence, usage practices, experiences and incentive structures; however, there are diverse opinions about their structural and content-related design. The quality of life is mentioned as an essential evaluation parameter for multimedia applications.

**Discussion:**

The highly variable individual life circumstances of MMD and their PAs, along with limited access options and a lack of suitability of the multimedia applications, may be causal factors for their relatively limited use, especially in domestic settings. Daily performance capabilities and individual disease progression pose special requirements for the scientific evaluation and the demonstration of the effectiveness of touch-based multimedia applications.

Die Zahl der Menschen mit Demenz (MMD) in Deutschland wird Prognosen zufolge im Jahr 2050 auf bis zu 2,35 Mio. ansteigen [3]. Etwa zwei Drittel der MMD werden dabei von pflegenden Angehörigen (PA) im häuslichen Setting (HS) gepflegt [21]. Da bislang noch keine medikamentöse Behandlung zur Heilung von Demenz existiert, wird auf personell aufwendige nichtmedikamentöse Therapieansätze zurückgriffen [5]. Der Einsatz touchbasierter Multimedia-Anwendungen im HS ist bislang gering ausgeprägt, weist aber Potenzial bei der kognitiven und körperlichen Aktivierung sowie psychosozialen Unterstützung von MMD und deren PA auf.

## Hintergrund

Demenzielle Erkrankungen sind einschneidende Lebensereignisse, die aufseiten der Betroffenen die Durchführung von Aufgaben des täglichen Lebens (ADL) in hohem Maße erschweren und auch deren PA mit neuen sozialen Rollen und psychosozialen Belastungen wie erhöhtem Depressionspotenzial, Einsamkeit und sozialer Isolation konfrontieren [14].

### Potenziale und Aufwände touchbasierter Multimedia-Anwendungen für MMD im HS

Spezielle touchbasierte Multimedia-Anwendungen können durch einfache Bedienung mit vergleichsweise wenig Vorbereitungsaufwand gezielt soziale, kognitive und körperliche Ressourcen durch themenbezogene affektive Stimulation aktivieren. Zielgruppengerechte Anwendungen haben so das Potenzial, zwischenmenschliche Interaktionen zu fördern und zu gesellschaftlicher Teilhabe anzuregen, was in einer subjektiv verbesserten Lebensqualität von MMD und ihren PA resultieren kann [19]. Nicht zuletzt aufgrund dieser wesentlichen Potenziale für die Betreuung und Unterstützung von MMD und ihren Pflegekräften werden touchbasierte Multimedia-Anwendungen im stationären Setting bereits vermehrt genutzt [14, 17]. Im Gegensatz zum stationären Setting stehen im HS aber nicht permanent (geschulte) Fachkräfte zur Verfügung, die die Einführung und Nutzung solcher Anwendungen unterstützen können. Dadurch wird ein Großteil der Betroffenen im HS nicht erreicht, die Passung verfügbarer Anwendungen für das HS ist nicht gegeben, und PA werden ggf. durch weitere Aufgaben überfordert. Die Motivation der PA ist im Kontext HS jedoch besonders relevant, da diese bereits in der Phase leichter neurokognitiver Störungen [1] – aber auch darüber hinaus – eine zentrale Rolle bei der Durchführung von ADL, einschließlich Beschäftigung und Aktivierung, einnehmen [17]. Die körperliche und geistige Gesundheit der PA stellt damit eine zentrale Voraussetzung für eine längerfristige Unterstützung von MMD und deren Verbleib in der Häuslichkeit dar [24].

### Wirtschaftlichkeit und Wirksamkeit touchbasierter Multimedia-Anwendungen für MMD im HS

Neben den kontextuellen und aufwandsbezogenen Faktoren spielen die wirtschaftlichen Faktoren eine nichtunwesentliche Rolle: Die in den jeweiligen App Stores bzw. Webshops der Hersteller vertriebenen kommerziellen touchbasierten Multimedia-Anwendungen zur Aktivierung von MMD im HS basieren zum einen meist auf einem (abobasierten) Selbstzahlermodell, und zum anderen ist eine nachweisliche Sicherung positiver Versorgungseffekte nicht verpflichtend – die Anwendungen sind so primär profitorientiert. Für Anwendungen im häuslichen Setting kommt hingegen auch eine Kostenerstattung durch die in der Pflegeversicherung als neue Leistungsform verankerten digitalen Pflegeanwendungen (DiPA) in Betracht. Hierfür sind allerdings wissenschaftliche Nachweise eines positiven Versorgungseffekts Voraussetzung. Ziel der DiPA ist es, die häusliche Versorgungssituation zu stabilisieren, PA zu entlasten, Selbstständigkeit zu erhalten und einer Zunahme der Pflegebedürftigkeit entgegenzuwirken. Es erscheint daher erforderlich, wissenschaftlich evaluierte touchbasierte Multimedia-Anwendungen speziell für das HS zu konzipieren und die Kriterien für potenzielle Entwickler*innen transparent zu machen. Individuelle Verlaufsunterschiede, individuelle soziale Umgebungen und wechselnde tagesformabhängige Verhaltenssymptome von MMD stellen in diesem Kontext Herausforderungen sowohl an die technisch-inhaltliche Konzeption als auch an die wissenschaftliche Evaluation multimedialer Anwendungen dar [19].

In diesem Beitrag werden Anforderungen, Einsatzmöglichkeiten sowie Gestaltungsoptionen touchbasierter Multimedia-Anwendungen zu Beschäftigung und Aktivierung für MMD und PA aus Expert*innensicht analysiert und diskutiert. Der Fokus liegt auf (A) Gestaltungsoptionen und Anforderungen an Anwendungsinhalte sowie (B) Anforderungen an Studiendesigns zu Wirksamkeitsnachweisen für oben genannte Technologien für MMD und ihre PA.

## Material und Methode

Im Rahmen der Untersuchung wurden Expert*innen mit mindestens 3‑jähriger praktischer Expertise zu Demenz (*n* = 7) bzw. wissenschaftlicher Erfahrung im Einsatz touchbasierter Multimedia-Anwendungen (*n* = 9) rekrutiert und mittels leitfadengestützter Einzelinterviews [9] einmalig online befragt. Die Themen des Leitfadens wurden anhand des theoretischen Vorverständnisses generiert (Zusatzmaterial online: Supplement 1).

Die Durchführung und Auswertung der Interviews ersteckten sich über den Zeitraum von Anfang Januar bis Ende Februar 2023. Insgesamt flossen 11 Expert*inneninterviews mit einer Dauer von 35–80 min in die Analyse ein.

Im Anschluss an die Volltranskription der Tonaufzeichnungen nach Dresing und Pehl [6] erfolgte die Auswertung mithilfe der computergestützten Daten- und Textanalyse Software MAXQDA (VERBI GmbH, Berlin) anhand einer strukturierenden Inhaltsanalyse nach Kuckartz [13]. Detaillierte Erläuterungen zum Sampling, zum Interviewleitfaden sowie zum Prozess der Datenauswertung: Zusatzmaterial online: Supplement 1.

## Ergebnisse

Die aus dem Vorverständnis gebildeten 3 Hauptkategorien (1. Einsatz digitaler Technologien bei Menschen mit Demenz, 2. Bedarfe an touchbasierte Multimedia-Anwendung aus Expert*innensicht, 3. Nutzungsszenarien und Anwendungsbedingungen) wurden gesamt auf 6 induktive Subkategorien erweitert (Zusatzmaterial online: Supplement 1); die Ergebnisse werden im Folgenden anhand von Ankerbeispielen dargestellt.

### Entwicklungsanforderungen

**Abb. 1:**
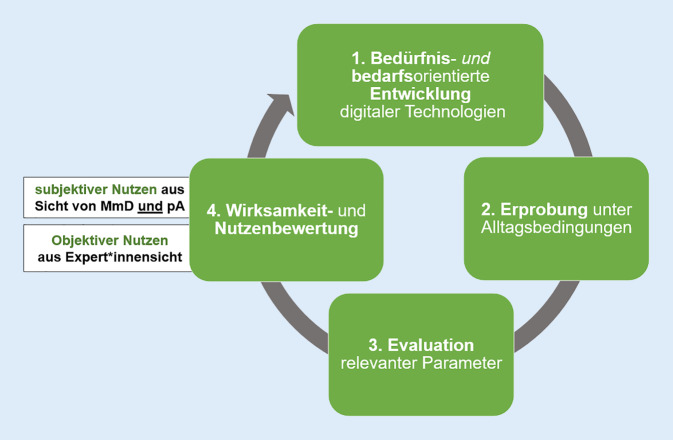
Entwicklungs- und Evaluationsprozess digitaler Anwendungen. (Eigene Abbildung)

Expert*innen stellen einstimmig die Wichtigkeit der frühen Einbindung Betroffener – sowohl der MMD als auch der PA – in den nutzendenzentrierten Entwicklungsprozess touchbasierter Multimedia-Anwendungen heraus (Abb. 1). Von Entwickler*innen eingeschätzte objektive Bedarfe bezüglich Design und Inhalt entsprechen laut Expert*innen nicht zwangsläufig den Bedürfnissen von MMD. Besonders kritisiert wird das defizitorientierte und pauschalisierende Fremdbild von Entwickler*innen, die MMD häufig innerhalb der Anwendungen „*als alte mit einem Faltenrock auftretende Damen am Kaffeetisch*“ (I3) assoziieren. Dies oder auch beispielsweise ein Vorspielen von Volksliedern sei nicht zeitgemäß und zielgruppengerecht. So berichtet eine Expertin z. B. über den Wunsch Betroffener nach Rock’n’Roll und „*fetziger*“ Musik. Es müsse „*validierend*“ auf die Menschen eingegangen werden, und die Diagnose „Demenz“ dürfe nicht zu Vorurteilen und Defizitorientierung führen.

### Nutzungskompetenzen und Akzeptanz

Als Voraussetzung für eine gelingende und zielführende Nutzung wird von Expert*innen die Notwendigkeit einer schrittweisen Heranführung an touchbasierte Multimedia-Anwendungen selbst sowie an die vermittelten Inhalte beschrieben. Hierfür benötige es *„einen Befähigungsrahmen“* (I3). Derzeit existiere laut mehreren Expert*innen meist Unwissen über die Existenz und Möglichkeiten solcher Anwendungen bei MMD und ihren PA.

Im Gegensatz dazu sind die Aussagen zur Technikaffinität der Zielgruppe sehr heterogen. Digitale Nutzungskompetenzen und -praktiken sowie bereits erworbene Erfahrungen im Umgang mit touchbasierten Multimedia-Anwendungen haben aus Sicht von Expert*innen einen entscheidenden Einfluss auf den Grad der einstellungsbezogenen Akzeptanz bzw. den Grad der Ablehnung digitaler Formate. Die Akzeptanz sei dabei alters- und generationenanhängig. Zudem korreliere ein höheres Bildungsniveau mit einer „*höheren Affinität*“ sowie einem „*höheren Verständnis für solche Geräte*“ (I6). Während ein Experte „*im großen Durchschnitt eher nichttechnikaffine Menschen*“ (I3) erlebt, beschreibt eine andere Expertin einen Wandel hin zu einer zunehmenden Nutzung touchbasierter Multimedia-Anwendungen und positiver Nutzungserfahrung. Eine Berücksichtigung und Einbeziehung bestehender sozialer Systeme und das Verständnis der Rollen von PA sind aus Sicht der Befragten grundlegend für die mehrwertbringende Nutzung digitaler Anwendungen.

Als unterstützende und motivierende Personen fungieren PA als „*kontinuierliche Ansprechpartner*“ (I2) und nehmen laut Expert*innen eine Schlüsselrolle in der Heranführung und der Sicherung einer nachhaltigen Durchführung sowie beim technischen Support ein.

### Anforderungen an Inhalte

#### Anreizstrukturen

Die Erwartung eines Nutzens beeinflusst aus Sicht der Befragten die Auseinandersetzung mit der Thematik und ist damit grundlegend für die Akzeptanz. Um die Nutzung zu fördern, wird auf die Wichtigkeit entsprechender Anreize hingewiesen. Eine „*Plüschummantelung, damit das Tablet sympathischer wird*“ (I3), zeigt den Versuch der Herstellung von Attraktivität. Möglichkeiten, digitale Anwendungen „*attraktiver zu machen“* und zu fördern, „*dass man sich besser und gerne damit auseinandersetzt*“ (I3), sehen Expert*innen neben der Integration körperlicher Aufgaben durch die Integration von Interaktionsmöglichkeiten, z. B. durch eine Spielfigur oder über die Möglichkeit einer gemeinsamen, kooperativen Nutzung mit PA. Auch leisten unterhaltende und gamifizierte Elemente sowie virtuelle Belohnungen, wie etwa Punktesysteme, aus Sicht mehrerer Befragter eine Hilfestellung zur Motivation. Andere Expert*innen betrachten das Nichterreichen von Leistungspunkten hingegen als mögliche Demotivation und raten daher von einem punktebasierten Belohnungssystem ab.

#### Individualisierung

Eine große Herausforderung touchbasierter Multimedia-Anwendungen liege in der situativen Anpassung auf das tagesaktuelle Leistungsniveau sowie der bedarfsorientierten Individualisierung von Inhalten. Unterschiedliche Krankheitsverläufe unterstreichen die Wichtigkeit, touchbasierte Multimedia-Anwendungen variabel und anpassungsfähig an das jeweilige Niveau Nutzender zu gestalten. Insbesondere nach der Diagnosestellung seien niederschwellige Angebote sinnvoll, da anfängliche Gedächtnisstörungen mit Scham und Verleugnung behaftet sein können.

#### Realitätsnahe, schlichte Darstellung

Expert*innen weisen auf die Wichtigkeit einer realitätsnahen Darstellung der Umgebung hin. Cartoons und illustrierte Figuren stellen beispielsweise eine zusätzliche kognitive Transferaufgabe dar, stören damit evtl. das Nutzungserleben und könnten stigmatisierend auf die Nutzenden wirken, da diese mit einer Infantilisierung assoziiert werden könnten. Weitere Anforderungen an die Darstellung sind Farbkontraste, maßstabsgetreue Darstellungen sowie komplexitätsreduzierte Auswahlmöglichkeiten. Visualisierungen werden wichtiger, je größer die Sprachbarriere ist, weshalb Expert*innen einer kombinierten audiovisuellen Stimulation einen hohen Wert zuschreiben.

### Wirkungen und Evaluation digitaler Technologien

#### Anforderung an Studiendurchführung

Das Design von Studien hat Einfluss auf die Generalisierbarkeit der Ergebnisse. Ein typischer Bias resultiere aus der Inklusion vorwiegend „*weißer Akademikerfamilien*“ (I9). Auch weisen freiwillig Teilnehmende nach Erfahrungen der Expert*innen oft eine überdurchschnittliche Gesundheitskompetenz auf, weshalb Gesundheitskompetenz und Bildung zusätzliche Einflussfaktoren darstellen, die berücksichtigt werden müssen. Eine möglichst hohe Diversität erhöhe die Reichweite der Ergebnisse. Insbesondere bei text- und bildbasierten Anwendungen sei die Inklusion von Menschen mit Sprachbarrieren besonders wichtig. Darüber hinaus beschreiben die Befragten einen rein experimentellen Charakter von Evaluationsstudien als wenig zielführend, da er praxisfern sei und nicht die Komplexität der Realität abbilde. Auch werde eine regelmäßige, freiwillige Nutzung nach Beendigung des Studienzeitraums laut Expert*innen oft nicht erreicht.

#### Evaluation

Hier wird die besondere Schwierigkeit des Wirksamkeitsnachweises – u. a. mangels valider Evaluationskriterien – betont. Insbesondere für die Anerkennung als DiPA und die damit einhergehende Erhöhung der Reichweite sei dieser aber entscheidend. Während in der klinischen Forschung quantitative Nachweise als Goldstandard gelten, beschreibt eine Expertin dieses Vorgehen für MMD als „*schwierig, weil das von einem sehr linearen Wirkungsmodell ausgeht, was an sich schon nicht mehr zeitgemäß ist und bei interaktiven Medienangeboten schon mal gar nicht“ *(I8). Von mehreren Expert*innen wird angezweifelt, dass durch die Technologienutzung signifikante Verbesserungen in standardisierten Tests, wie beispielsweise dem Mini-Mental-Status-Test (MMST), zu erreichen sind.

Wohlbefinden und Lebensqualität werden als übergeordnete relevante Parameter zur Evaluation touchbasierter Multimedia-Anwendungen genannt. Unabhängig von einer Verbesserung kognitiver Fähigkeiten stünden „*Lebensqualität, Würde und der Spaßfaktor an erster Stelle“* (I10). Positives Erleben wird auch von einer anderen Expertin herausgestellt: „*wenn es Spaß macht, dann hat es auch einen Nutzen*“ (I9). Spaß wissenschaftlich zu evaluieren, sei sehr herausfordernd, jedoch ein relevanter Aspekt bezüglich Zuwendung, Einstellung und Nutzung touchbasierter Multimedia-Anwendungen, der nicht vernachlässigt werden sollte.

Eine weitere Möglichkeit der qualitativen Evaluation ergebe sich in der Analyse der Beziehungsqualität und Kommunikation von MMD und PA. Während einige Expert*innen gemeinsames Spielen mit einer positiven Beziehungsgestaltung assoziieren, könnte eine gemeinsame Nutzung auch zu Scham bei Betroffenen führen. Die offensichtliche Hilfsbedürftigkeit durch die Asymmetrie in der kognitiven Leistungsfähigkeit von MMD vs. PA könnte sich verstärkt offenbaren und damit die Beziehungsgestaltung stören. Diesbezüglich solle untersucht werden, ob durch die Technologie mehr gemeinsame Zeit verbracht wird, und wie Betroffene und PA die Qualität der gemeinsamen Zeit bewerten, wenn die Anwendung gemeinsam genutzt wird.

## Diskussion

### Individuelle Anpassung an MMD

Sowohl die vorliegenden Studienergebnisse als auch die Literatur weisen auf die Wichtigkeit der individuellen Anpassung an das jeweilige Leistungsniveau Nutzender hin [19]. Technikaffinität scheint nicht nur altersabhängig, sondern auch sozialisationsbedingt zu sein, wobei hochaltrige Männer im Vergleich zu hochaltrigen Frauen signifikant positivere Technikeinstellungen aufweisen [18]. Neben der Affinität stellen, deckend zu den im Rahmen der Studie gewonnenen Erkenntnissen, eine schrittweise Heranführung an touchbasierte Multimedia-Anwendungen sowie die Akzeptanz Bedingungen für eine regelmäßige, freiwillige Nutzung dar [12]. Welche Nutzungskompetenzen und Leistungsdifferenzierungen den Betroffenen durch Expert*innen zugeschrieben werden, variiert jedoch und beeinflusst Anforderungen an die Hardware und Software touchbasierter Multimedia-Systeme.

### Zielgruppengerechte Designlösungen

Diesbezüglich weisen die im „REAFF“-Framework (*R*eactive, *E*nabling, *A*ugmenting, *F*ailure-*F*ree) vorgeschlagenen Grundsätze zur Entwicklung technologischer Lösungen für MMD [2] – in Verbindung mit dem hoch bewerteten Wunsch nach Anwendungen zur Beziehungsgestaltung in der „Demenz-Technologie-Wunschliste“ von Sixsmith et al. [20] sowie der Wichtigkeit bedürfnisgerechter Lösungen – auf das Potenzial von „warm technology“ [11] hin. Diese hat zum Ziel, soziale Verbundenheit, Würde und das Selbstvertrauen zu unterstützen und eine Verbesserung der psychosozialen Lebensqualität zu erreichen. Übereinstimmend mit dem REAFF-Framework [2] sowie der Demenz-Technologie-Wunschliste erachten Expert*innen Warm technology durch ansprechende und nichtstigmatisierende Designlösungen als relevant [11].

### Exergames zur körperlichen Aktivierung

Die von Expert*innen beschriebenen Potenziale der Integration von Bewegungsaufgaben decken sich mit der Literatur [22, 23, 25, 26]. Beim sogenannten Exergaming („exercise gaming“) werden Bewegungsübungen – angeregt durch eine virtuelle, bildschirmbasierte Spielumgebung – durchgeführt, um positive physische, kognitive und emotionale Effekte zu erzielen [22, 23, 25]. Exergaming, das körperliches Training und kognitive Stimulation kombiniert, kann so Verbesserungen kognitiver und körperlicher Funktionen bei älteren MMD erwirken [25]. Studien hierzu weisen jedoch eine Heterogenität in Bezug auf Dauer und Häufigkeit der Nutzung innerhalb des Interventionszeitraums auf [23].

### Belastungsempfinden der PA und wissenschaftliche Evaluation touchbasierter Multimedia-Anwendungen

Dass digitale Mulimedia-Anwendungen allein keine „*Lösung*“ (I5) darstellen und deren Wirkungsreichweite begrenzt und derzeit nicht umfassend untersucht ist [16], unterstreicht die Wichtigkeit der Einbindung sozialer Systeme. PA haben hierbei über den gesamten Prozess von der Heranführung über die Durchführung bis hin zum technischen Support eine entscheidende Rolle inne, während sie gleichzeitig selbst verschiedenen Belastungen ausgesetzt sind [14]. Antworten auf die Fragen, inwieweit touchbasierte Mulimedia-Anwendungen dabei zu einer Entlastung Angehöriger führen und/oder ADL der Betroffenen erhalten und damit die Pflegebedürftigkeit verringern können, sind bislang offen. Bei der Analyse sollte zwischen zeitlicher Entlastung, pflegerischer und emotionaler Entlastung sowie einem Gewinn an Lebensqualität differenziert werden. Zwar existieren als „valide“ geltende Messverfahren zur Evaluation von Lebensqualität (z. B. SF-36, WHOQOL-OLD, H.I.L.D.E), jedoch ist Lebensqualität als multidimensionales Konstrukt aus physischen, psychischen und umweltbezogenen Dimensionen sowohl stark individuell als auch sozial konnotiert, komplex und dynamisch [15]. Die subjektive Bewertung der PA bezüglich der empfundenen Entlastung und der empfundenen Mehrwerte in Bezug auf Lebensqualität ist dabei entscheidend [12]; dies geht auch aus der vorliegenden Erhebung hervor.

### Kontextuelle Limitationen

Auch steht im Raum, wie sich eine mögliche Entlastung reliabel erfassen ließe, und welche Studiendesgins dafür geeignet sind. Herausfordernd gestaltet sich hierbei – deckend mit Einschätzungen der Expert*innen – eine klinische Validierung positiver Versorgungseffekte [7]. Komplexe Lebensrealitäten und verschiedene Störeinflüsse (soziales Umfeld, Krankheitsstadium und -Verlauf) sowie individuelle Bedürfnisse und heterogene Betroffenen-Angehörigen-Dyaden erschweren die Benennung konkreter Wirkungsketten und Nutzungsszenarien von touchbasierten Multimedia-Anwendungen im HS. Kausalitätsketten zwischen digitaler Intervention und Ergebnis sind dabei vielschichtig und langwierig [10]. Die meisten Interventionen werden in Laboren unter optimalen Bedingungen und nicht im HS durchgeführt [27]. Eine zu geringe Proband*innenzahl sowie ein nichtrandomisiertes Sampling stellen weitere Limitationen von Wirksamkeitsstudien bezüglich Multimedia-Anwendungen für MMD dar [8]. Darüber hinaus sind Follow-up-Studien nötig, um Aussagen zur freiwilligen Nutzung touchbasierter Multimedia-Anwendungen im HS über den Interventionszeitraum hinweg tätigen zu können.

### Co-Design zusammen mit der Zielgruppe

Die frühe Einbindung von MMD und ihren PA in die Entwicklung digitaler Aktivierungsinhalte stellt aus Sicht der Expert*innen daher die Grundvoraussetzung für eine freiwillige und anhaltende Nutzung – auch über den Interventionszeitraum von Studien – dar. Negative, defizitorientierte Fremdbilder von MMD können nur in direktem Kontakt mit der Zielgruppe aufgelöst werden, um bedürfnis- und ressourcenorientierte Inhalte zu entwickeln. Als praxisnaher Forschungsansatz bietet schließlich „citizen science“ die Möglichkeit einer partizipativen Forschung und Kooperation von Wissenschaftler*innen und Bürger*innen – sowohl PA als auch MMD –, um Impulse aus der Gesellschaft in die Forschung einfließen zu lassen [4].

## Limitationen der vorliegenden Studie

Es wurde keine bestehende touchbasierte Multimedia Technologie evaluiert. Durch die Befragung unabhängig Forschender konnte die Gefahr von sozial erwünschtem Antwortverhalten und interessengeleiteten Darlegungen zum Einsatz touchbasierter Multimedia-Anwendungen verringert werden. Die Befragung von Expert*innen mit jeweils sehr spezifischen, fachlichen Schwerpunkten schmälerte möglicherweise die Themendurchdringung im Einzelfall. Jedoch konnte so im Querschnitt ein erstes differenziertes Bild zu verschiedenen Aspekten des Untersuchungsgegenstands erlangt werden.

## Fazit für die Praxis

- Anpassungen an individuelle Bedürfnisse, Krankheitsverläufe und Technikaffinitäten sowie tagesabhängige Leistungsfähigkeiten innerhalb der Zielgruppe stellen zentrale Herausforderungen in der Konzeption digitaler Interventionen für MMD dar.
- Der Einsatz touchbasierter Multimedia-Anwendungen setzt einen Befähigungsrahmen und eine Einbettung in soziale Systeme voraus.
- PA spielen eine Schlüsselrolle bei der Motivation zur regelmäßigen Nutzung, weshalb deren Einbindung in die Konzeption und Entwicklung digitaler Interventionen unerlässlich ist.
- Einsatzmöglichkeiten touchbasierter Multimedia-Anwendungen sehen Expert*innen insbesondere, um das Wohlbefinden Betroffener zu verbessern und um die Interaktion und Kommunikation mit PA zu unterstützen.
- MMD und ihre PA sollten bereits von Anfang an aktiv in die Entwicklung von touchbasierter Multimedia-Anwendungen für das HS eigebunden werden, um bedürfnisorientierte Inhalte bereitstellen zu können.

### Supplementary Information

Erläuterung zum Studiendesign und zur Auswertungsmethode der Expert*inneninterviews
