# Supplementary material for: Design and evaluation options of multimedia applications to support the care of people with mild dementia
Source: Z Gerontol Geriatr. 2024 Jan 24;57(5):382–8. [Article in German] doi: 10.1007/s00391-023-02280-2 (PMC11315789; doi:10.1007/s00391-023-02280-2)
Supplement: Supplementary file 1 — Erläuterung zum Studiendesign und zur Auswertungsmethode der Expert*inneninterviews [file 391_2023_2280_MOESM1_ESM.docx]

**Supplement 1: Studiendesign und Methode**

Leitfadenerstellung:

Der Leitfaden für die Expert*inneninterviews wurde auf Grundlage einer vorherigen Literaturrecherche, des aktuellen Kenntnisstandes sowie zentralen Fragestellungen entwickelt. Er gliedert sich in die Dreiteilung [6]

(1) Einstiegsfragen,

(2) Hauptteil mit Frageblöcken zu Themen und Unterthemen

(3) Ausstieg und Dank.

Als vorab vereinbarte und systematisch angewandte Vorgabe zur Gestaltung des Interviewablaufs enthält der Leitfaden sowohl offene, als auch teiloffene Fragen zu jeweiligen Kategorien. Dabei folgte die Erstellung dem Prinzip „*So offen wie möglich, so strukturierend wie nötig*“. [3]

Rekrutierung und Sampling:

Expert*inneneninterviews zeichnen sich durch die Besonderheit der Zielgruppe aus [1]. Bei diesen werden Befragte aufgrund des ihnen zugeschriebenen Status als Expert*innen interviewt. [3] Dem Erkenntnisinteresse folgend, wurden folgende Einschlusskriterien festgelegt:

Expert: innen^[[1]](#footnote-1)^, die mindestens 3 Jahre Berufserfahrung aufweisen und eine der folgenden Kriterien erfüllen:

**Einschlusskriterien:**

Schwerpunkt Wissenschaft:

- Tätigkeit in öffentlicher Institution
- Wissenschaftliche Expertise an der Schnittstelle von Neurologie (Schwerpunkt Demenz), Neurotechnologie und Sozialwissenschaften.
- hierzu Öffentlich zugängliche Publikationsarbeit

Schwerpunkt Praxis:

- Kranken- oder Altenpfleger*in, Arzt/Ärztin oder Betreuungskraft
- min. dreijährige Erfahrung in der Zusammenarbeit mit Demenzpatient*innen,
- Tätigkeit in der häuslichen ambulanten Versorgung
- Weiterbildung im Bereich Digitalisierung

**Ausschlusskriterien:**

- Berufserfahrung unter drei Jahren
- Unternehmer*in mit Vertrieb eigener digitaler Anwendungen
- Anderweite wirtschaftliche Abhängigkeiten durch offensichtliche Kooperation mit Unternehmen dig. Anwendungen und damit Befangenheit
- Fehlende Expertise speziell mit Menschen mit Demenz

Tabelle 1: Soziodemografische Daten der teilnehmenden Expert*innen

| **Expert*in** | **Geschlecht** | **Expertise Wissenschaft** | **Expertise Praxis** |
| --- | --- | --- | --- |
| E 1 | w |  | x |
| E 2 | w | x | x |
| E 3 | w |  | x |
| E 4 | m | x | x |
| E 5 | m | x |  |
| E 6 | w | x | x |
| E 7 | w | x |  |
| E 8 | m | x |  |
| E 9 | w | x |  |
| E 10 | w | x | x |
| E 11 | w | x | x |

Zunächst wurden, den Einschlusskriterien entsprechende Expert*innen, im Internet recherchiert und per Mail anhand eines Informationsschreibens kontaktiert. Bei Interesse erhielten die Expert*innen bei Bedarf weitere Informationen zum geplanten Ablauf des Gesprächs sowie technische Hinweise zur Durchführung. Alle Teilnehmenden wurden vorab über das Vorhaben und Vorgehen in Kenntnis gesetzt und gaben ihr schriftliches Einverständnis zur Durchführung des Online-Interviews.

Aus dem theoretischen Vorverständnis und der Festlegung deduktiv gebildeter Kategorien ergab sich die Strukturierung des Fragebogens.

**HK1****. Einsatz digitaler Technologien bei Menschen mit Demenz**

SK1: Einstellung und Bewertung Nutzender
SK2: Nutzungskompetenzen

**HK 2****:** **Bedarfe an touchbasierte Multimedia- Anwendung aus Expert*innensicht**

SK1: Anforderungen an Entwicklung
SK2: Anforderungen an Inhalte
SK3: Anforderungen an Design
SK4: Anforderung an Studiendurchführung und wissenschaftliche Evaluation

**HK3: Nutzungsszenarien und Anwendungsbedingungen**

Tabelle 2. Interview-Leitfaden – Expert*innen

| **Hauptkategorie** | **Subkategorien** | **Vertiefung Themenfeld und Fragestellung** |
| --- | --- | --- |
| **HK1. Allgemeines zum Einsatz digitaler Technologien bei Menschen mit Demenz** | SK1: Einstellung und Bewertung  SK2: Nutzungskompetenzen | **Bewertung** **Zielgruppe auf** ***emotionaler Ebene****:*   - Wie erleben Sie die Akzeptanz von Demenzpatient*innen ggü. digitalen tabletbasierten Mulitmedia-Technologien? - Wie erleben Sie die Nutzungskompetenzen von Demenzpatient*innen ggü. digitalen tabletbasierten Technologien? - Von welchen Faktoren sind diese Nutzungskompetenzen abhängig? |
| **Konkretisierung** |  |  |
| **HK 2: Bedarfe aus Expert*innensicht**  Wie können digitale Anwendungen **Bedarfe** aufgreifen? | - 1. Anforderungen an Entwicklung   2. Anforderungen Anwendungsinhalt   3. Anforderungen Technologiegestaltung   4. Wissenschaftliche Evaluation digitaler Technologien (Wirksamkeitsuntersuchung, Operationalisierung) | - Was ist ihres Erachtens bei der Entwicklung dig. tabletbasierter Anwendungen für MmD im häuslichen Setting zu berücksichtigen? - Was ist ihrer Meinung nach in der inhaltlichen Konzeption dig. Beschäftigungs- und Aktivierungsangebote zu beachten? - Inwieweit können digitale Anwendungen zum  a) Erhalt der Selbständigkeit, Lebensführung und Alltagsgestaltung, - b) Kongitionsverbesserung/Erhalt von Demenzbetroffenen beitragen? - Welche Anforderungen bestehen bezüglich   a) Gebrauchstauglichkeit und Alltagstauglichkeit b) Design   - Wie kann eine wissenschaftliche Evaluierung tabletbasierter Anwendungen erfolgen? - Welche Endparameter erachten Sie im Bereich der Beschäftigung und Aktivierung als relevant? |
| **HK3**: Nutzungsszenarien und  Anwendungsbedingungen | Nutzungsszenarien und  Anwendungsbedingungen | 1. Welche umweltbezogenen Bedingungen (gemäß ICF) sind in Bezug auf die Nutzung relevant? 2. Welche Rolle spielen Angehörige in der Durchführung von digitalen Anwendungen? 3. (wie) Können digitale Anwendungen ihrer Erfahrung nach zu einer Entlastung pflegender Angehöriger führen? |

Analyse:

Die Auswertung und Durchführung der Einzelinterviews liefen in einem iterativen Prozess über einen Zeitraum von Januar bis Ende Februar 2023. Es wurden so viele Interviews geführt, bis in Bezug auf die auf dem Leitfaden hervorgehenden Themen eine theoretische Sättigung erreicht wurde. Insgesamt flossen elf Expert:inneninterviews mit einer Dauer zwischen 35 und 80 Minuten in die Analyse ein.

Anschließend an die Volltranskription des qualitativen Datenmaterials nach inhaltlich semantischen Transkriptionsregeln von Dressing und Pehl [2], erfolgte die Auswertung des empirischen Materials anhand einer strukturierenden Inhaltsanalyse nach Kuckartz (Abb.1) [4][5]. Im Mittelpunkt der qualitativen Inhaltsanalyse standen dabei die Kategorien, die sowohl deduktiv als auch induktiv gebildet wurden. Inhaltlich relevante Teile des Interviews wurden Haupt- und Subkategorien zugeordnet, wobei das gesamte Datenmaterial systematisch durchgearbeitet und codiert wurde.[5] Zur qualitativen Daten- und Textanalyse wurde die Software MAXQDA genutzt. In MAXQDA wurde ein Kodierleitfaden angerfertigt, aus dem die Definition gebildeter Kategorien hervorgeht. Diese wurden jeweils mit Ankerbeispielen illustriert. Insgesamt wurden 241 Codes gebildet.


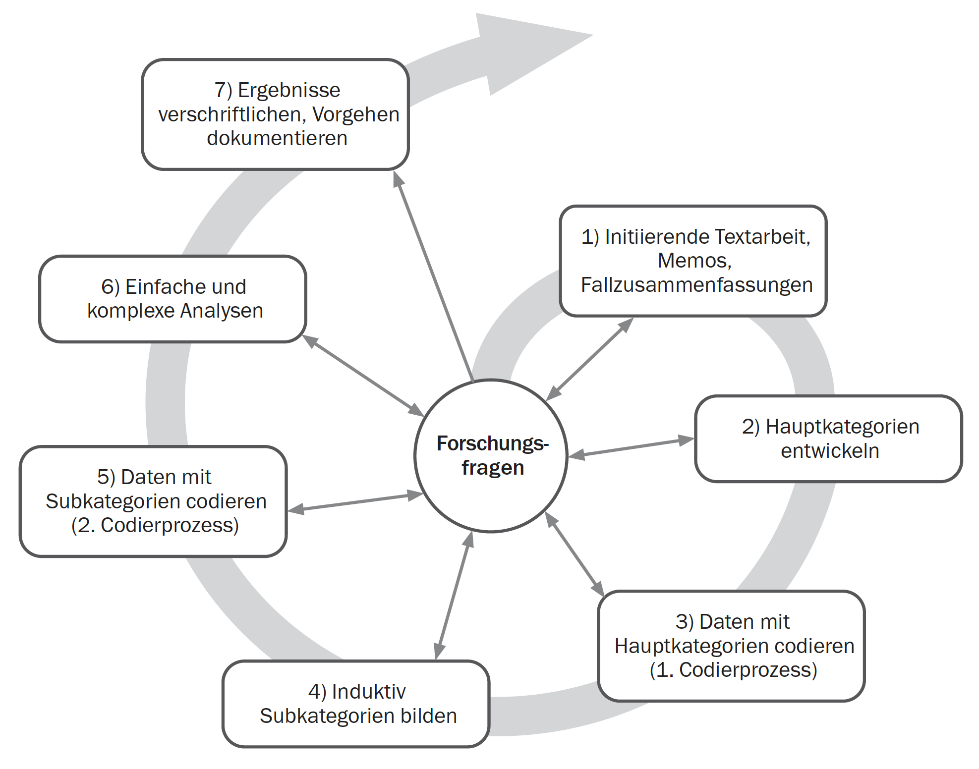


*Abb. 1: Ablauf einer inhaltlich strukturierenden qualitativen Inhaltsanalyse in 7 Phasen In: Qualitative Inhaltsanalyse. Methoden, Praxis, Computerunterstützung“ (Kuckartz & Rädiker, 2022, S.132)*

Deduktive Subkategorien wurden durch aus dem empirischen Material gebildeten induktiven Kategorien erweitert. Es wurden folgende *induktive Kategorien* hinzugefügt:

HK1, SK2: Nutzungskompetenzen: *2.1 Alter, 2.2 Geschlecht, 2.3 Technikaffinität, 2.4 Erfahrungsraum*

Hk2: SK 2.3 Wissenschaftliche Evaluation digitaler Technologien: 2*.3.1 Sampling, 2.3.2 Studiendurchführung 2.3.3 Evaluationsparamater*

Eine interne Validierung der Analyse des Materials erfolgte durch das Hinzuziehen eines zweiten Forschers (4-Augen-Prinzip) und steigerte die Gültigkeit der Ergebnisse.

Tabelle 3. Kodierleitfaden Expert*inneninterviews

| **Kategorie** |  | **Definition** | **Ankerbeispiel** | **Kodierregel** |
| --- | --- | --- | --- | --- |
| Allgemeines zum Einsatz digitaler Technologien bei Menschen mit Demenz | SK1: Einstellung und Bewertung | Alle Stellen, in denen Allgemeines zur Einstellung zum Einsatz digitaler Technologien bei Menschen mit Demenz definiert wird | *Das hängt von den digitalen Nutzungspraktiken ab, den Nutzungserfahrungen, der Nutzungskompetenz. Und letztendlich auch ein Stück weit mit der Passung des Angebots. Also ob die App einen funktionalen Mehrwert hat für die Betroffenen. (I8, Abs.2)* | *Nur jene Stellen sie sich mit Einstellungen im Spektrum zwischen Ablehnung und Akzeptanz von MmD ggü. Dig. Technologien befassen* |
|  | SK2: Nutzungskompetenzen | Alle Stellen, in denen Nutzungskompetenzen von MmD zum Einsatz digitaler Technologien auf mobilen Endgeräten beschriebenen werden | *„es kommt immer grundsätzlich ganz drauf an, wo kommt jemand her, wo ist er aufgewachsen, wo hat er gewohnt?“ I2, Abs.3).* | *Nur jene Stellen sie sich auf Alter, Geschlecht, Technikaffinität, oder Erfahrungsraum vom MmD mit dig. Technologien auf mobilen Endgeräten befassen* |
| HK 2: Bedarfe aus Expert*innensicht | SK1: Anforderungen an Entwicklung | Alle Stellen, in denen Anforderungen an die Entwicklung von Aktivierungssystemen für MmD und pA beschriebenen werden | *Sobald man partizipativ arbeitet und den Leuten die es benutzen sollen die Chance gibt, es mitzugestalten ist eine sehr gute Basis geschaffen“. (I3, Abs. 65)* | Nur jene Stellen, die bei der Entwicklung dig. tabletbasierter Anwendungen für MmD im häuslichen Setting zu berücksichtigen sind |
|  | SK2: Anforderungen an Inhalte | Alle Stellen, in denen Anforderungen an die Inhalte und themenspezifische Ausgestaltungen von Aktivierungssystemen für MmD und pA beschriebenen werden | *„man kann alle möglichen Alltagssituationen nehmen, an denen die Nutzenden anknüpfen können, das ist natürlich biografieabhängig, aber es gibt ja einiges, womit sich viele Menschen gerne beschäftigen, wie Reisen, Tiere oder Essen.“ (I1, Abs. 5)* | Nur jene Stellen, die bei der inhaltlichen Ausgestaltung dig. tabletbasierter Anwendungen für MmD im häuslichen Setting zu berücksichtigen sind |
|  | SK3: Anforderungen an Design und Hartware | Alle Stellen, in denen Anforderungen an Soft- und Hardware von Aktivierungssystemen für MmD und pA beschriebenen werden | *„reduzierte Auswahlmöglichkeiten müssen gegeben sein, um Reizüberflutung zu vermeiden.“ (I3, Abs.4)* | Nur jene Stellen, die sich auf die Hard- und Software dig. tabletbasierter Anwendungen für MmD im häuslichen Setting zu beziehen. Dau gehörigen Design, die Medienpräsentation und Bedienbarkeit. |
|  | SK4: Anforderung an Sampling, Studiendurchführung und wissenschaftliche Evaluation |  | *Sampling: in den Kontexten in den ich unterwegs bin, sowohl in der Klinik als auch in den Forschungsprojekten sowieso schon einen gewissen Selection Bias gibt, Auswahl Bias. Das man dann tendenziell eher infomiertere Leute, Leute die sich um ihre eigene Gesundheit kümmern (I6, Pos.2)*  Evaluationsparamater*: Das ist natürlich richtig schwierig, weil da müssen sie sich überlegen wie wäre der Verlauf, wenn es diese Interaktion nicht gäbe? Also sie haben keine Kontrollgruppe. Geht ja in dem Fall nicht. Also wie oft schaut sich ein Mensch mit Demenz eine Anwendung an und macht mit. Und vielleicht auch wie steigt das Aufmerksamkeitsniveau im Vergleich. Am wichtigsten ist aber Lebensqualität. (I10, Pos.3)* | *Nur jene Stelle, die sich auf Sampling, Studiendurchführung und Evaluationsparamater*  tabletbasierter Anwendungen für MmD und ihre pA im hS beziehen. |
| HK3: Nutzungsszenarien und Anwendungsbedingungen |  | Nutzungsszenarien und  Anwendungsbedingungen | *Wenn der Mensch mit Demenz weniger Erfahrung hat, wäre es ja erstmal die Rolle der Angehörigen zunächst mal an solche Digitalisierungsinstrumente heranzuführen und dann natürlich auch zu begleiten (I4, Pos.2)* | Nut jene Stellen , die sich auf Nutzungsszenarien und  Anwendungsbedingungen dig. Aktivierungssysteme auf dem Tablet für MmD in der Häuslichkeit beziehen. |

**Literatur**

1. Ahlrichs R (2012) Zwischen sozialer Verantwortung und ökonomischer Vernunft. Springer VS, Wiesbaden

2. Dresing T, Pehl T, Hrsg. (2018) Praxisbuch Transkription, 2. Aufl. Dr. Dresing und Pehl GmbH, Marburg

3. Helfferich C (2019) Leitfaden- und Experteninterviews. In: Baur N, Blasius J (Hrsg.) Handbuch Methoden der empirischen Sozialforschung. Springer Fachmedien Wiesbaden, Wiesbaden, S. 669–686

4. Kuckartz U (2018) Qualitative Inhaltsanalyse. Methoden, Praxis, Computerunterstützung, 4. Aufl. Beltz, Weinheim

5. Kuckartz U, Rädiker S (2022) Datenaufbereitung und Datenbereinigung in der qualitativen Sozialforschung. In: Baur N, Blasius J (Hrsg.) Handbuch Methoden der empirischen Sozialforschung, 3. Aufl. Springer VS, Wiesbaden, S. 501–516

6. Mieg HA, Brunner B (2001) Experteninterviews: eine Einführung und Anleitung. ETH Zurich

1. Der Begriff Experte beschreibt die spezifische Rolle des/der Interviewpartner*in als Quelle von Spezialwissen über zu erforschende Sachverhalte [1] [↑](#footnote-ref-1)
